# (2*R*,3*R*,4*S*,5*R*)-2-(4-Amino-5-iodo-7*H*-pyrrolo­[2,3-*d*]pyrimidin-7-yl)-5-methyl­tetra­hydro­furan-3,4-diol

**DOI:** 10.1107/S1600536813027931

**Published:** 2013-10-16

**Authors:** Ulrich Flörke, Birte Drewes

**Affiliations:** a Department Chemie, Fakultät für Naturwissenschaften, Universität Paderborn, Warburgerstrasse 100, D-33098 Paderborn, Germany; b Institut für Neurowissenschaften und Medizin, Nuklearchemie (INM-5), Forschungszentrum Jülich GmbH, D-52428 Jülich, Germany

## Abstract

The mol­ecular structure of the title compound, C_11_H_13_IN_4_O_3_, shows a ribo­furanos­yl–pyrrolo O—C—N—C torsion angle of 59.1 (3)°, with the central C—N bond length being 1.446 (3) Å. The C—I bond length is 2.072 (2) Å. The amino group is coplanar with the attached aromatic ring [C—N—C—N torsion angle = −178.8 (2)°] and forms an intra­molecular N—H⋯I hydrogen bond. In the crystal, O—H⋯N and N—H⋯O hydrogen bonds link the mol­ecules into puckered layers parallel to (001). These layers are bound to each other by secondary I⋯O inter­actions [3.2250 (17) Å], forming a three-dimensional framework.

## Related literature

For background to the use of marine natural products as therapeutic agents, see: Kazlauskas *et al.* (1983 ▶); Mitchell *et al.* (1996 ▶); Wiesner *et al.* (1999 ▶); Ugarkar *et al.* (2000 ▶); Song *et al.* (2011 ▶). For the structures of related compounds, see: Seela *et al.* (1996 ▶, 1999 ▶, 2008 ▶).
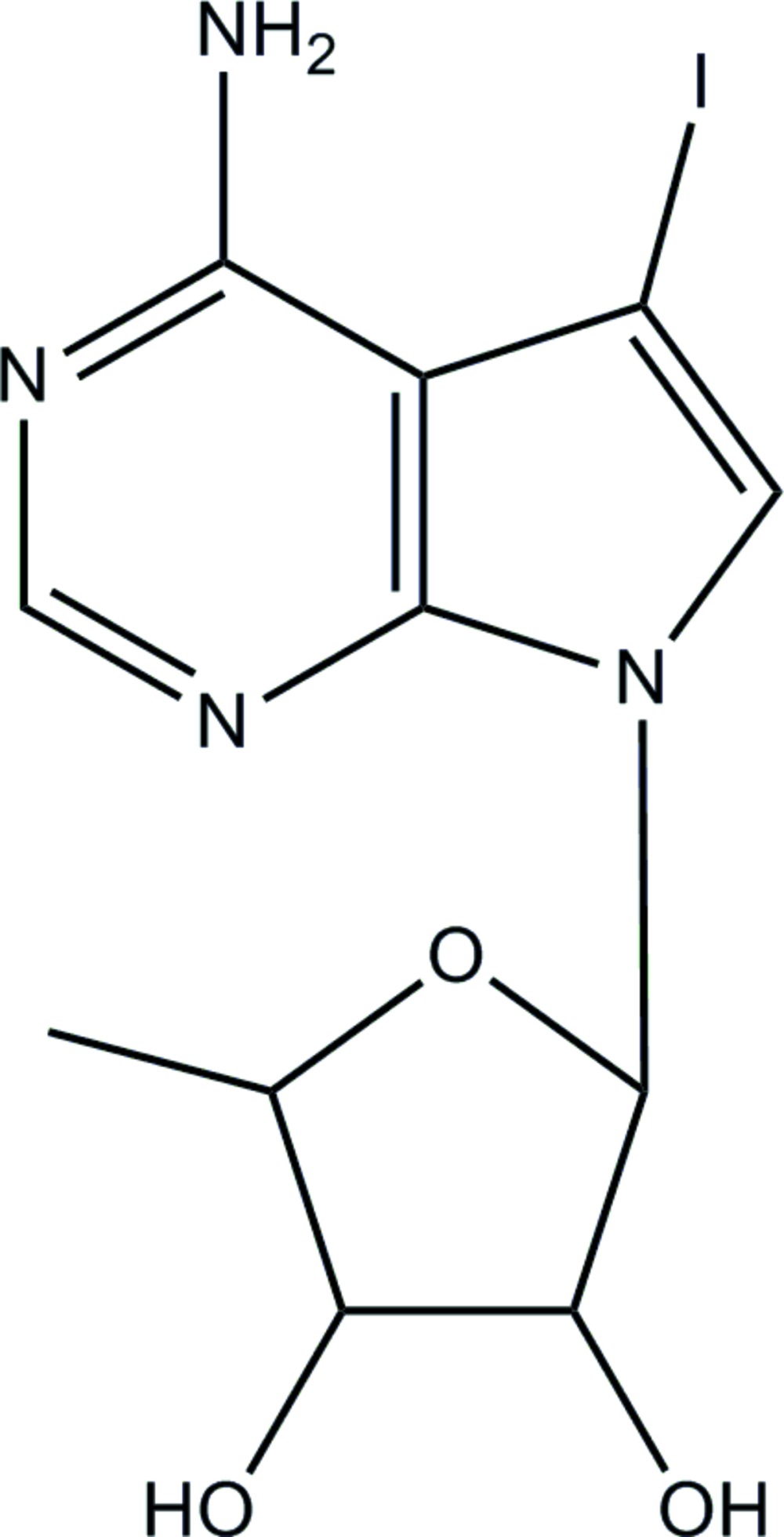


## Experimental

### 

#### Crystal data

C_11_H_13_IN_4_O_3_
*M* *_r_* = 376.15
Orthorhombic, 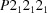

*a* = 4.9164 (2) Å
*b* = 14.6490 (5) Å
*c* = 18.0130 (6) Å
*V* = 1297.30 (8) Å^3^
*Z* = 4
Mo *K*α radiation
μ = 2.48 mm^−1^
*T* = 130 K
0.49 × 0.08 × 0.08 mm

#### Data collection

Bruker SMART APEX diffractometer
Absorption correction: multi-scan (*SADABS*; Sheldrick, 2004 ▶) *T*_min_ = 0.376, *T*_max_ = 0.826
12307 measured reflections
3103 independent reflections
3056 reflections with *I* > 2σ(*I*)
*R*_int_ = 0.019

#### Refinement

*R*[*F* ^2^ > 2σ(*F* ^2^)] = 0.020
*wR*(*F* ^2^) = 0.051
*S* = 1.06
3103 reflections
175 parameters
H-atom parameters constrained
Δρ_max_ = 1.05 e Å^−3^
Δρ_min_ = −0.30 e Å^−3^
Absolute structure: Flack (1983 ▶), 1274 Friedel pairs
Absolute structure parameter: −0.015 (17)

### 

Data collection: *SMART* (Bruker, 2002 ▶); cell refinement: *SAINT* (Bruker, 2002 ▶); data reduction: *SAINT*; program(s) used to solve structure: *SHELXTL* (Sheldrick, 2008 ▶); program(s) used to refine structure: *SHELXTL*; molecular graphics: *SHELXTL*; software used to prepare material for publication: *SHELXTL* and local programs.

## Supplementary Material

Crystal structure: contains datablock(s) global, I. DOI: 10.1107/S1600536813027931/kq2009sup1.cif

Structure factors: contains datablock(s) I. DOI: 10.1107/S1600536813027931/kq2009Isup2.hkl

Supplementary material file. DOI: 10.1107/S1600536813027931/kq2009Isup3.cml

Additional supplementary materials:  crystallographic information; 3D view; checkCIF report

## Figures and Tables

**Table 1:** Hydrogen-bond geometry (Å, °)

*D*—H⋯*A*	*D*—H	H⋯*A*	*D*⋯*A*	*D*—H⋯*A*
O2—H2⋯N2^i^	0.84	1.94	2.759 (3)	165
O3—H3⋯N1^ii^	0.84	2.16	2.907 (3)	149
N4—H4*A*⋯O2^iii^	0.88	2.07	2.915 (3)	160
N4—H4*B*⋯I1	0.88	2.92	3.636 (2)	139

Symmetry codes: (i) 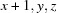; (ii) 
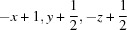
; (iii) 
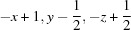
.

**Fig. 1.**
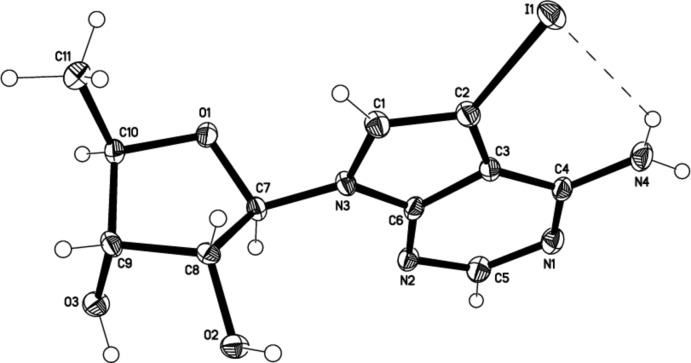
Molecular structure of the title compound with anisotropic displacement parameters drawn at the 50% probability level. The intramolecular N4–H4b···I1 hydrogen bond depicted as dashed line.

**Fig. 2.**
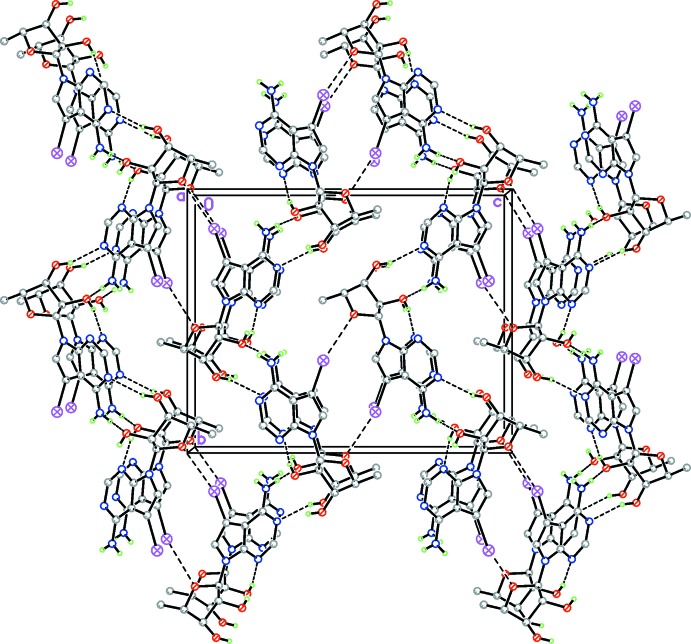
Crystal packing viewed along a axis with intermolecular I···O interaction as well as hydrogen bonds as dashed lines.

## supplementary crystallographic information

### 1. Comment

Marine natural products provide a rich source of chemical diversity that can be used to develop new, potentially useful therapeutic agents. Nucleosides from marine organisms show great potential as lead compounds in medicinal chemistry research. 5'-Deoxy-5-iodotubercidin (5'd-5IT, 4-amino-5-iodo-7-(5-deoxy-β-*D*-ribofuranosyl)pyrrolo[2,3-*d*]pyrimidine) was isolated from the marine red alga Hypnea vanlendiae (Kazlauskas *et al.*, 1983) and from the marine ascidian Didemnum voeltzkowi (Mitchell *et al.*, 1996). In vitro, all 5'-deoxytubercidin marine nucleosides show strong inhibitory activity for human adenosine kinase with 5'd-5IT being the most potent one. Therapeutic success of adenosine kinase inhibitors as active agents in animal models is documented for epilepsy and pain and as antiseizure agents (Wiesner *et al.*, 1999; Ugarkar *et al.*, 2000). The molecular structure is related to the derivatives studied previously (Seela *et al.*, 1996, 1999, 2008) and shows no unexpected geometric parameters.

### 2. Experimental

The title compound was synthesized according to a known procedure (Song *et al.*, 2011). Recrystallization from ethanol-water (1:1) yielded crystals suitable for X-ray analysis. Spectroscopic analysis: ^1^H NMR (250 MHz, DMSO-d6, δ): 1.29 (d, J = 6.16 Hz, 3H, CHCH_3_), 3.87–3.95 [m, 2H, H-3', H-4'], 4.41 (m, 1H, H-2'), 5.11 (s, 1H, 3'-OH), 5.33 (s, 1H, 2'-OH), 6.02 (d, J = 5.28 Hz, 1H, H-1'), 6.69 (s, 2H, 4-NH_2_), 7.63 (s, 1H, H-6), 8.14 (s, 1H, H-2); ^13^C NMR (250 MHz, DMSO-d6, δ): 19.6 (CH_3_, C-5'), 52.8 (C—I, C-5), 73.8 (CH, C-2'), 75.0 (CH, C-3'), 79.8 (CH, C-4'), 87.4 (CH, C-1'), 103.6 (C═C, C-4a), 127.4 (CH, C-6), 150.8 (C═C, C-7a), 152.5 (CH, C-2), 157.6 (C—NH_2_, C-4).

### 3. Refinement

Hydrogen atoms were clearly identified in difference syntheses, refined at idealized positions riding on the carbon, nitrogen or oxygen atoms with C—H 0.95–1.00, N—H 0.88, O—H 0.84 Å and with isotropic displacement parameters *U*_iso_(H) = 1.2*U*_eq_(C/N) or 1.5*U*_eq_(—CH_3_ and —OH H atoms). All CH_3_ and OH hydrogen atoms were allowed to rotate but not to tip. The max. electron density residual is close (0.9 Å) to the I1 position.

### Crystal data

**Table:**

C_11_H_13_IN_4_O_3_	*F*(000) = 736
*M**_r_* = 376.15	*D*_x_ = 1.926 Mg m^−^^3^
Orthorhombic, *P*2_1_2_1_2_1_	Mo *K*α radiation, λ = 0.71073 Å
Hall symbol: P 2ac 2ab	Cell parameters from 8237 reflections
*a* = 4.9164 (2) Å	θ = 2.7–28.3°
*b* = 14.6490 (5) Å	µ = 2.48 mm^−^^1^
*c* = 18.0130 (6) Å	*T* = 130 K
*V* = 1297.30 (8) Å^3^	Needle, colourless
*Z* = 4	0.49 × 0.08 × 0.08 mm

### Data collection

**Table:**

Bruker SMART APEX diffractometer	3103 independent reflections
Radiation source: sealed tube	3056 reflections with *I* > 2σ(*I*)
Graphite monochromator	*R*_int_ = 0.019
φ and ω scans	θ_max_ = 27.9°, θ_min_ = 1.8°
Absorption correction: multi-scan (*SADABS*; Sheldrick, 2004)	*h* = −6→6
*T*_min_ = 0.376, *T*_max_ = 0.826	*k* = −19→19
12307 measured reflections	*l* = −23→22

### Refinement

**Table:**

Refinement on *F*^2^	Secondary atom site location: difference Fourier map
Least-squares matrix: full	Hydrogen site location: difference Fourier map
*R*[*F*^2^ > 2σ(*F*^2^)] = 0.020	H-atom parameters constrained
*wR*(*F*^2^) = 0.051	*w* = 1/[σ^2^(*F*_o_^2^) + (0.0319*P*)^2^ + 0.5639*P*] where *P* = (*F*_o_^2^ + 2*F*_c_^2^)/3
*S* = 1.06	(Δ/σ)_max_ = 0.001
3103 reflections	Δρ_max_ = 1.05 e Å^−^^3^
175 parameters	Δρ_min_ = −0.30 e Å^−^^3^
0 restraints	Absolute structure: Flack (1983), 1274 Friedel pairs
Primary atom site location: structure-invariant direct methods	Absolute structure parameter: −0.015 (17)

### Special details

**Table:**

Geometry. All e.s.d.'s (except the e.s.d. in the dihedral angle between two l.s. planes) are estimated using the full covariance matrix. The cell e.s.d.'s are taken into account individually in the estimation of e.s.d.'s in distances, angles and torsion angles; correlations between e.s.d.'s in cell parameters are only used when they are defined by crystal symmetry. An approximate (isotropic) treatment of cell e.s.d.'s is used for estimating e.s.d.'s involving l.s. planes.
Refinement. Refinement of *F*^2^ against ALL reflections. The weighted *R*-factor *wR* and goodness of fit *S* are based on *F*^2^, conventional *R*-factors *R* are based on *F*, with *F* set to zero for negative *F*^2^. The threshold expression of *F*^2^ > σ(*F*^2^) is used only for calculating *R*-factors(gt) *etc*. and is not relevant to the choice of reflections for refinement. *R*-factors based on *F*^2^ are statistically about twice as large as those based on *F*, and *R*- factors based on ALL data will be even larger.

### Fractional atomic coordinates and isotropic or equivalent isotropic displacement parameters (Å^2^)

**Table:**

	*x*	*y*	*z*	*U*_iso_*/*U*_eq_	
I1	0.89717 (3)	0.148805 (10)	0.080859 (9)	0.02262 (6)	
O1	0.5927 (4)	0.52637 (11)	0.01354 (9)	0.0194 (3)	
O2	1.0140 (4)	0.58588 (12)	0.16890 (10)	0.0197 (4)	
H2	1.1212	0.5464	0.1858	0.030*	
O3	0.7492 (4)	0.71478 (11)	0.08382 (10)	0.0228 (4)	
H3	0.8160	0.7236	0.1261	0.034*	
N1	0.2124 (5)	0.28946 (15)	0.26704 (12)	0.0224 (4)	
N2	0.3108 (4)	0.43351 (14)	0.20816 (11)	0.0189 (4)	
N3	0.6561 (4)	0.42430 (13)	0.11225 (11)	0.0162 (4)	
N4	0.4171 (5)	0.15312 (15)	0.23436 (11)	0.0243 (4)	
H4A	0.3194	0.1255	0.2686	0.029*	
H4B	0.5318	0.1215	0.2070	0.029*	
C1	0.8108 (5)	0.35459 (17)	0.08162 (13)	0.0192 (4)	
H1A	0.9408	0.3619	0.0430	0.023*	
C2	0.7476 (5)	0.27404 (16)	0.11546 (14)	0.0191 (5)	
C3	0.5438 (5)	0.29313 (16)	0.17014 (13)	0.0160 (5)	
C4	0.3910 (6)	0.24402 (16)	0.22372 (12)	0.0185 (5)	
C5	0.1823 (5)	0.37938 (18)	0.25567 (14)	0.0223 (5)	
H5A	0.0501	0.4085	0.2860	0.027*	
C6	0.4924 (5)	0.38688 (16)	0.16605 (13)	0.0164 (5)	
C7	0.6628 (5)	0.51898 (15)	0.08971 (13)	0.0154 (4)	
H7A	0.5302	0.5547	0.1202	0.018*	
C8	0.9424 (5)	0.56288 (15)	0.09505 (12)	0.0150 (5)	
H8A	1.0830	0.5217	0.0730	0.018*	
C9	0.9025 (5)	0.64656 (15)	0.04619 (12)	0.0178 (4)	
H9A	1.0796	0.6710	0.0274	0.021*	
C10	0.7272 (5)	0.60780 (16)	−0.01690 (13)	0.0182 (5)	
H10A	0.5861	0.6538	−0.0312	0.022*	
C11	0.8868 (6)	0.58060 (18)	−0.08480 (14)	0.0274 (5)	
H11A	1.0502	0.5471	−0.0697	0.041*	
H11B	0.9398	0.6355	−0.1124	0.041*	
H11C	0.7742	0.5415	−0.1165	0.041*	

### Atomic displacement parameters (Å^2^)

**Table:**

	*U*^11^	*U*^22^	*U*^33^	*U*^12^	*U*^13^	*U*^23^
I1	0.02487 (8)	0.01568 (8)	0.02731 (8)	0.00340 (6)	−0.00081 (7)	−0.00292 (6)
O1	0.0223 (8)	0.0186 (8)	0.0174 (7)	−0.0041 (7)	−0.0021 (8)	0.0033 (6)
O2	0.0225 (9)	0.0172 (8)	0.0193 (8)	0.0048 (7)	−0.0047 (7)	−0.0027 (7)
O3	0.0320 (10)	0.0158 (7)	0.0207 (8)	0.0065 (7)	−0.0005 (9)	−0.0020 (7)
N1	0.0230 (11)	0.0239 (10)	0.0203 (10)	−0.0025 (9)	0.0016 (9)	0.0048 (8)
N2	0.0217 (10)	0.0181 (10)	0.0170 (9)	0.0027 (8)	0.0005 (8)	0.0030 (8)
N3	0.0177 (11)	0.0120 (9)	0.0191 (9)	0.0006 (7)	0.0022 (8)	0.0007 (7)
N4	0.0286 (11)	0.0227 (10)	0.0216 (10)	−0.0061 (12)	0.0010 (9)	0.0076 (8)
C1	0.0192 (10)	0.0170 (10)	0.0213 (10)	0.0002 (9)	0.0033 (9)	−0.0001 (11)
C2	0.0207 (13)	0.0160 (11)	0.0206 (11)	0.0005 (9)	−0.0014 (10)	0.0000 (9)
C3	0.0162 (12)	0.0145 (10)	0.0174 (10)	−0.0010 (8)	−0.0018 (9)	0.0019 (8)
C4	0.0209 (12)	0.0172 (10)	0.0175 (10)	−0.0019 (10)	−0.0055 (11)	0.0043 (8)
C5	0.0231 (12)	0.0227 (11)	0.0210 (12)	0.0039 (10)	0.0030 (10)	0.0017 (9)
C6	0.0167 (10)	0.0174 (11)	0.0149 (10)	−0.0008 (9)	−0.0026 (9)	0.0042 (9)
C7	0.0150 (10)	0.0130 (9)	0.0182 (11)	−0.0019 (8)	−0.0009 (9)	0.0020 (8)
C8	0.0154 (12)	0.0135 (10)	0.0161 (11)	0.0006 (8)	0.0007 (8)	−0.0024 (8)
C9	0.0205 (10)	0.0116 (9)	0.0212 (10)	−0.0014 (12)	0.0032 (9)	−0.0001 (8)
C10	0.0233 (12)	0.0136 (10)	0.0177 (11)	−0.0011 (9)	0.0018 (10)	0.0027 (9)
C11	0.0375 (14)	0.0256 (12)	0.0190 (11)	−0.0042 (12)	0.0058 (15)	0.0001 (10)

### Geometric parameters (Å, º)

**Table:**

I1—C2	2.072 (2)	C1—C2	1.364 (3)
O1—C7	1.419 (3)	C1—H1A	0.9500
O1—C10	1.470 (3)	C2—C3	1.433 (4)
O2—C8	1.417 (3)	C3—C6	1.398 (3)
O2—H2	0.8400	C3—C4	1.419 (3)
O3—C9	1.423 (3)	C5—H5A	0.9500
O3—H3	0.8400	C7—C8	1.521 (3)
N1—C5	1.341 (3)	C7—H7A	1.0000
N1—C4	1.350 (3)	C8—C9	1.522 (3)
N2—C5	1.327 (3)	C8—H8A	1.0000
N2—C6	1.356 (3)	C9—C10	1.535 (3)
N3—C6	1.374 (3)	C9—H9A	1.0000
N3—C1	1.388 (3)	C10—C11	1.507 (3)
N3—C7	1.446 (3)	C10—H10A	1.0000
N4—C4	1.351 (3)	C11—H11A	0.9800
N4—H4A	0.8800	C11—H11B	0.9800
N4—H4B	0.8800	C11—H11C	0.9800
			
C7—O1—C10	108.28 (17)	O1—C7—C8	104.37 (18)
C8—O2—H2	109.5	N3—C7—C8	114.11 (19)
C9—O3—H3	109.5	O1—C7—H7A	109.5
C5—N1—C4	117.9 (2)	N3—C7—H7A	109.5
C5—N2—C6	111.9 (2)	C8—C7—H7A	109.5
C6—N3—C1	107.93 (19)	O2—C8—C7	112.61 (19)
C6—N3—C7	126.5 (2)	O2—C8—C9	112.55 (18)
C1—N3—C7	125.6 (2)	C7—C8—C9	100.81 (18)
C4—N4—H4A	120.0	O2—C8—H8A	110.2
C4—N4—H4B	120.0	C7—C8—H8A	110.2
H4A—N4—H4B	120.0	C9—C8—H8A	110.2
C2—C1—N3	109.5 (2)	O3—C9—C8	111.00 (18)
C2—C1—H1A	125.2	O3—C9—C10	108.4 (2)
N3—C1—H1A	125.2	C8—C9—C10	101.68 (18)
C1—C2—C3	107.3 (2)	O3—C9—H9A	111.8
C1—C2—I1	123.41 (19)	C8—C9—H9A	111.8
C3—C2—I1	128.88 (18)	C10—C9—H9A	111.8
C6—C3—C4	116.0 (2)	O1—C10—C11	108.81 (19)
C6—C3—C2	106.4 (2)	O1—C10—C9	106.05 (18)
C4—C3—C2	137.6 (2)	C11—C10—C9	114.0 (2)
N1—C4—N4	117.7 (2)	O1—C10—H10A	109.3
N1—C4—C3	119.2 (2)	C11—C10—H10A	109.3
N4—C4—C3	123.1 (2)	C9—C10—H10A	109.3
N2—C5—N1	129.3 (2)	C10—C11—H11A	109.5
N2—C5—H5A	115.4	C10—C11—H11B	109.5
N1—C5—H5A	115.4	H11A—C11—H11B	109.5
N2—C6—N3	125.4 (2)	C10—C11—H11C	109.5
N2—C6—C3	125.7 (2)	H11A—C11—H11C	109.5
N3—C6—C3	108.9 (2)	H11B—C11—H11C	109.5
O1—C7—N3	109.84 (18)		
			
C6—N3—C1—C2	−0.2 (3)	C2—C3—C6—N2	179.6 (2)
C7—N3—C1—C2	−178.7 (2)	C4—C3—C6—N3	179.7 (2)
N3—C1—C2—C3	−0.1 (3)	C2—C3—C6—N3	−0.4 (3)
N3—C1—C2—I1	173.35 (17)	C10—O1—C7—N3	−151.81 (19)
C1—C2—C3—C6	0.3 (3)	C10—O1—C7—C8	−29.1 (2)
I1—C2—C3—C6	−172.66 (19)	C6—N3—C7—O1	−119.2 (2)
C1—C2—C3—C4	−179.9 (3)	C1—N3—C7—O1	59.1 (3)
I1—C2—C3—C4	7.2 (5)	C6—N3—C7—C8	124.0 (2)
C5—N1—C4—N4	−178.8 (2)	C1—N3—C7—C8	−57.7 (3)
C5—N1—C4—C3	1.9 (4)	O1—C7—C8—O2	162.92 (18)
C6—C3—C4—N1	−0.8 (3)	N3—C7—C8—O2	−77.2 (2)
C2—C3—C4—N1	179.3 (3)	O1—C7—C8—C9	42.8 (2)
C6—C3—C4—N4	179.9 (2)	N3—C7—C8—C9	162.66 (19)
C2—C3—C4—N4	0.1 (5)	O2—C8—C9—O3	−44.0 (3)
C6—N2—C5—N1	1.1 (4)	C7—C8—C9—O3	76.2 (2)
C4—N1—C5—N2	−2.2 (4)	O2—C8—C9—C10	−159.10 (19)
C5—N2—C6—N3	−179.8 (2)	C7—C8—C9—C10	−38.9 (2)
C5—N2—C6—C3	0.2 (4)	C7—O1—C10—C11	126.7 (2)
C1—N3—C6—N2	−179.6 (2)	C7—O1—C10—C9	3.7 (2)
C7—N3—C6—N2	−1.1 (4)	O3—C9—C10—O1	−94.2 (2)
C1—N3—C6—C3	0.3 (3)	C8—C9—C10—O1	22.8 (2)
C7—N3—C6—C3	178.9 (2)	O3—C9—C10—C11	146.1 (2)
C4—C3—C6—N2	−0.3 (4)	C8—C9—C10—C11	−96.9 (2)

### Hydrogen-bond geometry (Å, º)

**Table:**

*D*—H···*A*	*D*—H	H···*A*	*D*···*A*	*D*—H···*A*
O2—H2···N2^i^	0.84	1.94	2.759 (3)	165
O3—H3···N1^ii^	0.84	2.16	2.907 (3)	149
N4—H4*A*···O2^iii^	0.88	2.07	2.915 (3)	160
N4—H4*B*···I1	0.88	2.92	3.636 (2)	139

Symmetry codes: (i) *x*+1, *y*, *z*; (ii) −*x*+1, *y*+1/2, −*z*+1/2; (iii) −*x*+1, *y*−1/2, −*z*+1/2.
